# Effect of Autologous Platelet Rich Fibrin on the Healing of Experimental Articular Cartilage Defects of the Knee in an Animal Model

**DOI:** 10.1155/2014/486436

**Published:** 2014-06-17

**Authors:** Davoud Kazemi, Ashraf Fakhrjou, Vahid Mirzazadeh Dizaji, Majid Khanzadeh Alishahi

**Affiliations:** ^1^Department of Veterinary Clinical Sciences, College of Veterinary Medicine, Tabriz Branch, Islamic Azad University, Tabriz, Iran; ^2^Department of Pathology, Faculty of Medicine, Tabriz University of Medical Sciences, Tabriz, Iran; ^3^College of Veterinary Medicine, Tabriz Branch, Islamic Azad University, Tabriz, Iran

## Abstract

The effect of autologous platelet rich fibrin (PRF), a second generation platelet product, on the healing of experimental articular cartilage lesions was evaluated in an animal model. Full thickness cartilage lesions with a diameter of 6 mm and depth of 5 mm were created in the weight bearing area of femoral condyles of both hind limbs in 12 adult mixed breed dogs. Defects in the left hind limb of each dog were repaired by PRF implantation whereas those in the right hind limb were left empty. The animals were euthanized at 4, 16, and 24 weeks following surgery and the resultant repair tissue was investigated macroscopically and microscopically. The results of macroscopic and histological evaluations indicated that there were significant differences between the PRF treated and untreated defects. In conclusion, the present study indicated that the use of platelet rich fibrin as a source of autologous growth factors leads to improvement in articular cartilage repair.

## 1. Introduction

Although articular cartilage injuries are among the most frequently encountered orthopaedic problems of the knee joint, their treatment has always been challenging for clinicians. The avascular nature of hyaline articular cartilage along with the low number of chondrocytes present and their minimum mitotic activity means that this particular connective tissue has a limited capacity for self-repair particularly in partial thickness injuries. The resultant repair tissue with its inferior biochemical and biomechanical characteristics ultimately fails, leading to the development of osteoarthritis or degenerative joint disease [1–4]. Therefore, all the treatment methods of articular cartilage injuries aim to produce a repair tissue with acceptable structural and functional properties in the long term [5].

Tissue engineering and the use of bioactive agents, that is, growth factors and cytokines, are the latest methods which are being employed for the treatment of articular cartilage injuries. Studies have indicated that a number of growth factors, namely, transforming growth factor *β*, basic fibroblast growth factor, insulin-like growth factor I, vascular endothelial growth factor, and platelet derived growth factor, have a positive influence in the repair and regeneration of hyaline cartilage. However there are limitations with the use of these products mainly due to high costs, short preservation period, and limited clinical availability. The aforementioned growth factors are released from the alpha granules of activated platelets following injury and the process of inflammation and tissue repair is initiated [6, 7]. Therefore various forms of platelet concentrates can be considered as rich source of autologous growth factors and could be used as an alternative to commercially available products [4, 8–10].

Platelet rich fibrin (PRF) is a second generation platelet concentrate which has many advantages over the first generation platelet rich plasma (PRP). PRF is produced by collecting autologous blood in glass tubes without any anticoagulant and immediate centrifugation. The resultant product is a true biomaterial containing fibrin clot, platelets, and leukocytes with a high concentration of growth factors. PRF does not require any activation prior to use and the growth factors are released slowly over a sustained period of time [11].

The aim of the present study was to investigate the effect of PRF as an autologous source of growth factors on healing of full thickness articular cartilage defects of the knee in an experimental animal model and to determine whether PRF use would have a positive effect on cartilage regeneration. For this purpose, macroscopic and microscopic characteristics of the repair tissue obtained following the use of PRF in articular cartilage defects were investigated.

## 2. Materials and Methods

Twelve skeletally mature mixed breed male dogs with the body weight of 20–30 kg were used in this study. The study was approved by the Experimentation Ethics Committee and Research Council of the Faculty of Veterinary Medicine, Islamic Azad University, Tabriz branch, and the animals were kept under the institutional laws for animal experiments.

The dogs were judged to be healthy based on the findings from physical examination and laboratory tests (complete blood cell count, blood biochemistry profiles, and urinalysis). The stifle joint (equivalent of the human knee) of each animal was carefully examined to rule out any joint instability. Skeletal maturity was determined by radiography prior to start of the experiment.

A total of 48 articular cartilage defects were created on the femoral condyles of the stifle joint (4 defects per dog). Defects in the left hind limb of each dog were repaired by PRF implantation whereas those in the right hind limb were left empty and considered as the controls.

### 2.1. PRF Preparation

**Figure 1:**
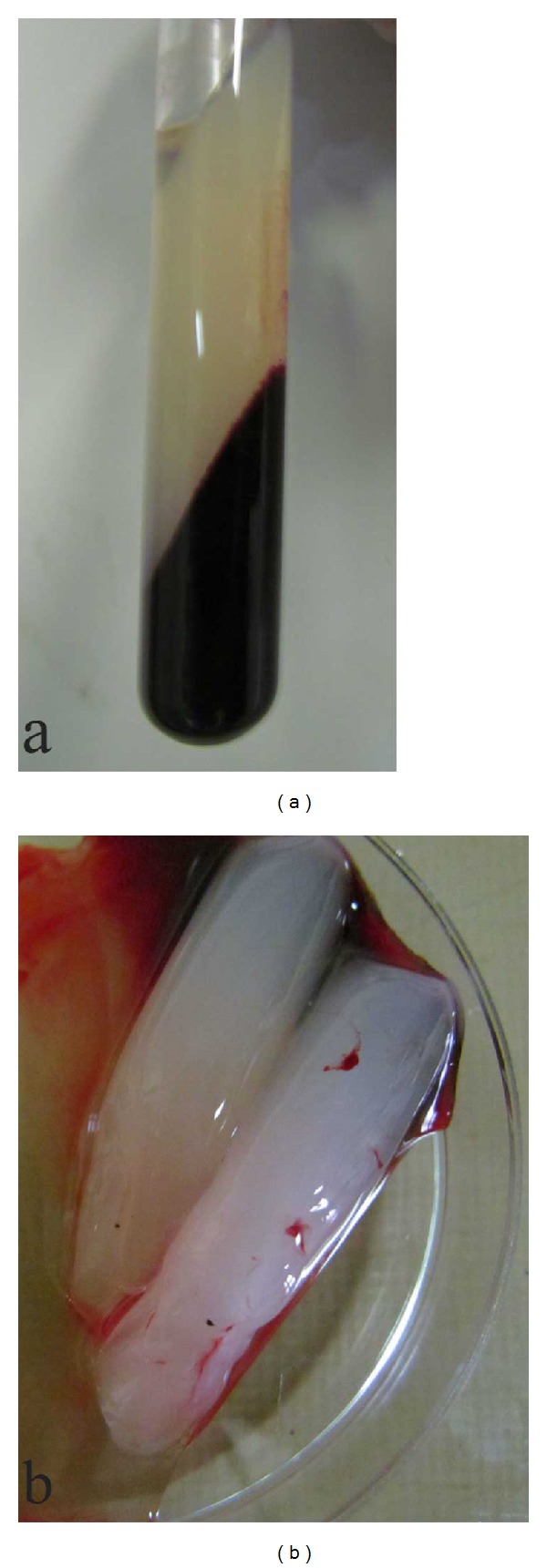
Venous blood sample following centrifugation indicating the PRF clot in the middle layer of the test tube (a) and the removed clots placed inside sterile petri dish (b).

Autologous PRF was prepared according to the method described by Dohan et al. [12]. Following premedication and prior to induction of anesthesia, 20 mL of blood was drawn from the jugular vein of each animal and collected in 10 mL sterile glass test tubes without any anticoagulant. The blood sample was centrifuged at 3000 rpm (400 g) for 10 minutes using a laboratory centrifuge. The resultant PRF clot which was located in the middle layer of the sample (Figure 1(a)) was removed from the test tube, and the red blood cells at the bottom and acellular plasma at the top of the tube were discarded. The PRF clots were kept in sterile petri dishes until later use during surgery (Figure 1(b)).

### 2.2. Surgical Procedure

**Figure 2:**
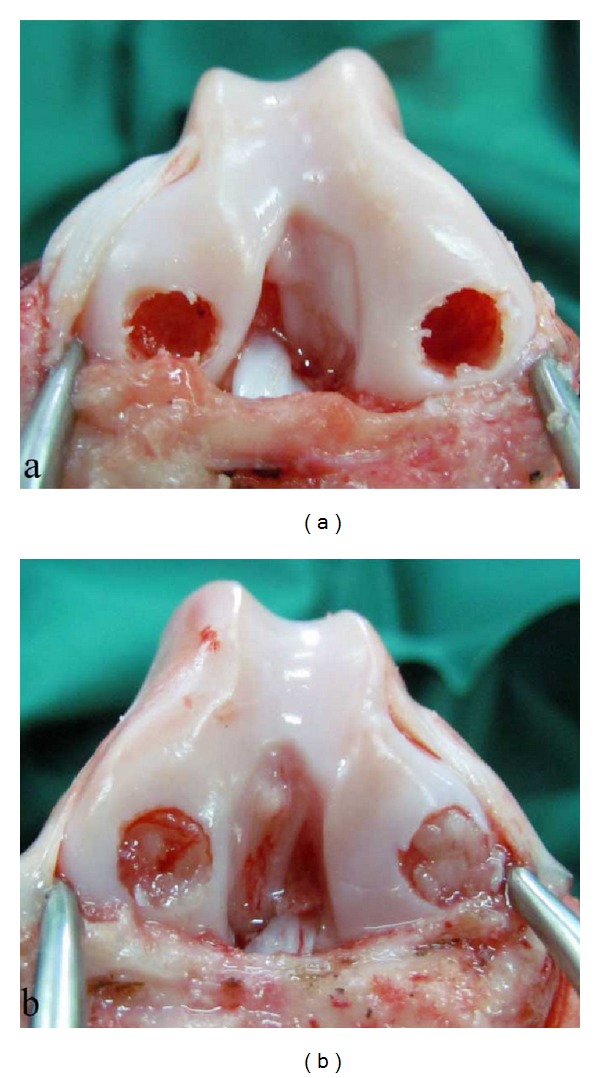
Full thickness articular cartilage defects left empty in the control group (a) and press-fitted with PRF clot in the treatment group (b).

Food was withheld from the animals for 12 hours before the surgery. Each dog was premedicated by intramuscular injection of xylazine (1 mg/kg) and atropine (0.04 mg/kg). Anesthesia was induced by intravenous injection of 2.5% solution of thiopental and maintained with halothane in oxygen following endotracheal intubation. Cefazolin (20 mg/kg) was given as preoperative antibiotic immediately following induction and lactated ringer's solution (10 mL/kg/hr) was infused during the surgery. The animal was placed in dorsal recumbency and under aseptic conditions; the medial approach to the stifle joint with lateral patellar luxation was used to gain access inside the joint. The joint was fully flexed to gain access to the weight bearing areas of the femoral condyles. Full thickness articular cartilage defects with a diameter of 6 mm and depth of 5 mm were created in the weight bearing area of each femoral condyle using a drill equipped with 6 mm drill bit. Bleeding was observed in all the defects confirming the involvement of subchondral bone and full thickness nature of the injury. Defects in the left stifle of dogs (*n* = 24) were repaired by implantation of PRF. Prior to PRF implantation, the defects on the lateral and medial condyles of the left stifle joint were lavaged thoroughly with normal saline solution. Each PRF clot prepared before the start of surgery was cut in half and the bottom half containing the maximum number of platelets was press-fitted inside each defect to completely fill the region (Figure 2(b)). Defects in the right stifle (*n* = 24) were lavaged and left empty without any treatment (Figure 2(a)). After completion of the procedure, the patella was returned to its normal position and the joint capsule, subcutaneous tissues, and skin were sutured routinely to close the wound. Postoperatively, penicillin (40000 IU/kg for 5 days) and ketoprofen (2.2 mg/kg for 3 days) were administered to the dogs. The animals were allowed to walk freely without any restrictions following recovery. Full weight bearing was allowed as tolerated by the dogs.

### 2.3. Sampling Procedure and Outcome Measurement

At 4, 16, and 24 weeks following surgery, the dogs were euthanized by an overdose of thiopental sodium injection and the distal femurs were harvested for macroscopic and histological evaluation of the repair tissue. Four dogs were randomly assigned to each of the sampling periods; therefore the number of PRF treated and control defects was 8 at each time period. Immediately after euthanasia, digital photographs of the defect area were taken and the International Cartilage Repair Society (ICRS) evaluation score [13] was used for macroscopic assessment of the repair tissue (Table 1).

Following macroscopic assessment, each femoral condyle was fixed in 10% buffered neutral formalin, decalcified, and embedded in paraffin for routine histological sectioning. Three sagittal sections (5 *μ*m thick) were cut from the centre of each defect and stained with haematoxylin-eosin and safranin-O/fast green and examined under the light microscope. Sections were blindly examined and scored according to the O'Driscoll histological grading scale [14] (Table 2). The mean score of the three sections was calculated for each sample.

### 2.4. Statistical Analysis

Comparison between the values obtained from the macroscopic and histological scores of PRF treated and control groups at each time point was made using the Mann-Whitney *U* test. To compare the macroscopic and microscopic scores at different time points within each group, the Kruskal-Wallis test followed by the post hoc Dunn's multiple comparison test was used. The significance level was set at 95% (*P* < 0.05). GraphPad Prism 5 software package (GraphPad Software Inc., La Jolla, CA) was used for data analysis.

## 3. Results

### 3.1. Macroscopic Observations

**Figure 3:**
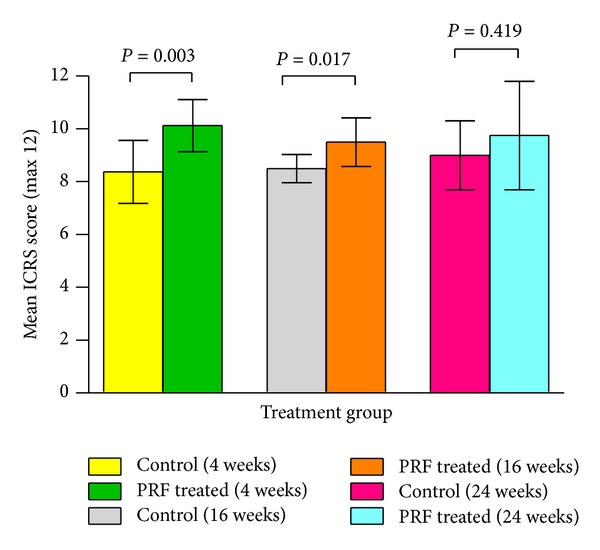
Mean ICRS scores for macroscopic evaluation of the repair tissue in the control and PRF treated groups at different time intervals. Error bars indicate standard deviation (SD) and the* P* values represent the statistical differences between the two treatment groups.

The distribution of mean ICRS scores between experimental groups at 3 different sampling times is shown in Figure 3. It is evident that the mean ICRS score of the control group is less than the PRF treated group at all sampling times and this difference is statistically significant (*P* < 0.05) at 4 and 16 weeks following surgery. Statistically significant differences in mean ICRS scores were not observed within each treatment group over time.

**Figure 4:**
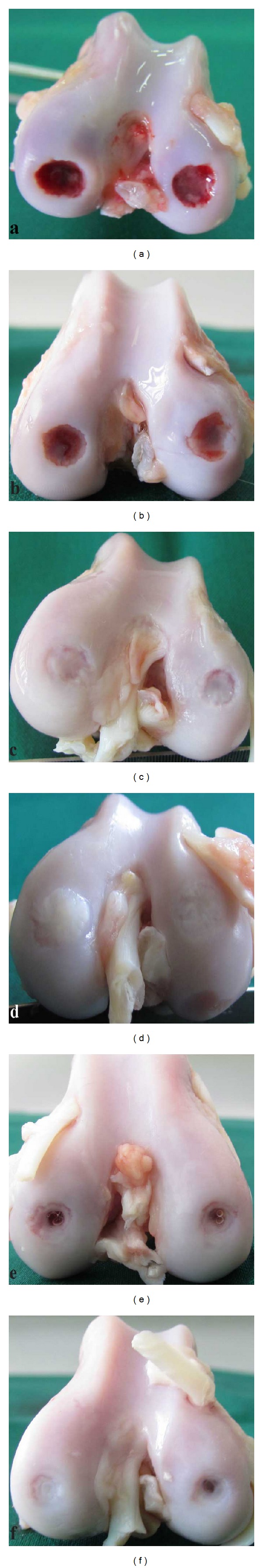
Macroscopic appearance of the representative defects from the two treatment groups at different time intervals: (a) control (4 weeks), (b) PRF treated (4 weeks), (c) control (16 weeks), (d) PRF treated (16 weeks), (e) control (24 weeks), and (f) PRF treated (24 weeks).

Four weeks after PRF implantation, the defects were filled with a bright red coloured fibrous repair tissue with discernible margins and white coloured areas resembling normal articular cartilage (Figure 4(b)). A central depression was also evident in the defects. The control defects were also filled with a quite similar repair tissue but the central depressions were larger and more evident than the PRF treated defects (Figure 4(a)).

At 16 weeks, the reparative tissue in both PRF treated and control groups were opaque white resembling normal surrounding cartilage (Figures 4(c) and 4(d)). The repair tissue had integrated well with the surrounding cartilage particularly in the PRF treated groups although the area of the defects could still be easily identified in both groups. The surface of the repair tissue was smoother in the PRF treated groups. Similar characteristics were observed at 24 weeks after surgery in both treatment groups although central cysts had developed inside the repair tissue (Figures 4(e) and 4(f)). The cystic cavities were larger and frequently encountered in the control group.

### 3.2. Histological Observations

**Figure 5:**
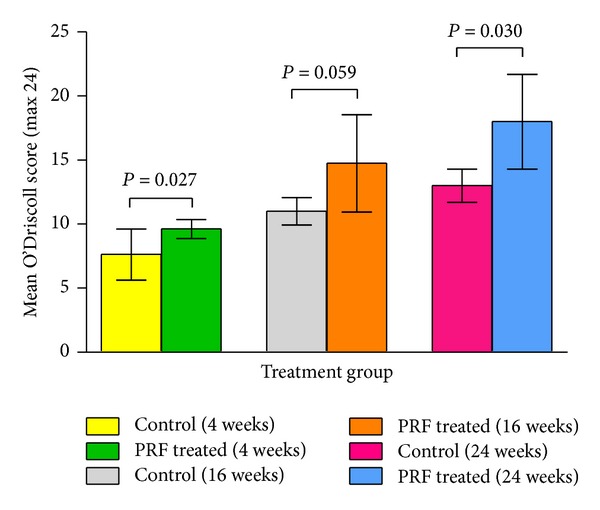
Mean O'Driscoll scores for histological evaluation of the repair tissue in the control and PRF treated groups at different time intervals. Error bars indicate standard deviation (SD) and the* P* values represent the statistical differences between the two treatment groups.

Mean histological scores of the two treatment groups at different postoperative times are shown in Figure 5. The O'Driscoll scores of PRF treated groups were higher than the control groups at all sampling times with significant differences (*P* < 0.05) observed at 4 and 24 weeks postoperatively. An increase in mean O'Driscoll scores was observed over time in both treatment groups. Within-group analysis of the histological results indicated that the mean O'Driscoll scores at 4 weeks were significantly (*P* < 0.05) lower compared to 16 and 24 weeks in both treatment groups but there was no statistically significant difference (*P* > 0.05) between 16 and 24 weeks.

**Figure 6:**
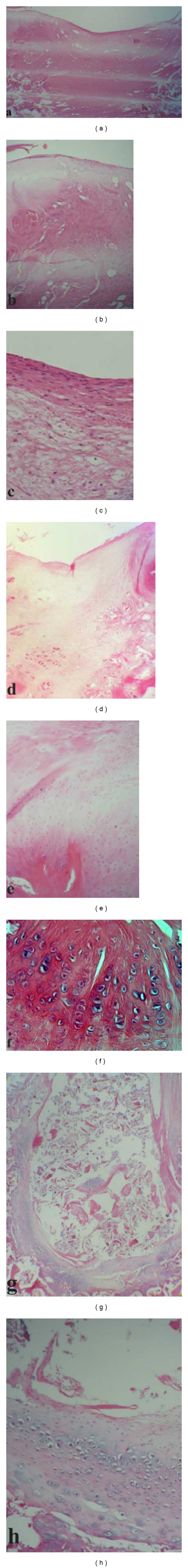
Histologic sections of the representative defects from the control group at different time intervals: 4 weeks ((a)–(c)), 16 weeks ((d)–(f)), and 24 weeks ((g) and (h)). Haematoxylin-eosin staining; original magnification: 40x ((a), (d), and (g)), 100x ((b), (e), and (h)), and 400x ((c) and (f)).

**Figure 7:**
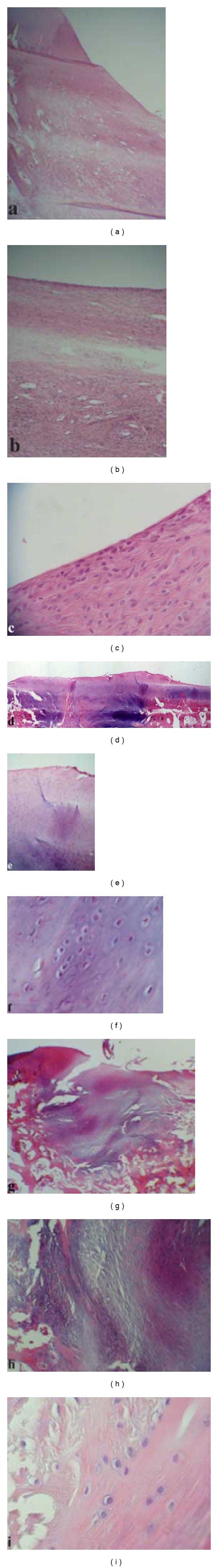
Histologic sections of the representative defects from the PRF treated group at different time intervals: 4 weeks ((a)–(c)), 16 weeks ((d)–(f)), and 24 weeks ((g)–(i)). Haematoxylin-eosin staining; original magnification: 40x ((a), (d), and (g)), 100x ((b), (e), and (h)), and 400x ((c), (f), and (i)).

Control and PRF treated defects were filled with fibrous tissue with abundant fibroblasts 4 weeks after surgery. Numerous blood vessels along with small cystic cavities were also observed in the repair tissue. In the control group, the cysts and blood vessels were much closer to the defect surface and more abundant than the PRF treated group. The repair tissue had bonded well with the surrounding normal cartilage in both groups. The surface of the defects was covered by fibroblasts in both groups although the surface was much smoother in the PRF treated group. No obvious evidence of cartilage-like tissue formation was observed in the repair tissue of both treatment groups (Figures 6(a)–6(c) and 7(a)–7(c)).

At 16 weeks, the number of fibroblastic cells in the repair tissue of the control defects had decreased and blood vessels were only observed in the deeper parts of the defect towards the subchondral bone area (Figure 6(d)). Chondrocyte-like oval cells inside lacunae were present at the edges of the defects where the repair tissue had bonded with the surrounding normal cartilage. The cells were arranged in columns emanating from the deeper parts of the defect towards the surface (Figures 6(e) and 6(f)). Similar changes in the histological characteristics of the repair tissue were also observed in the PRF treated defects although it was evident that the newly formed chondrocyte-like cells were distributed in almost all areas of the defect from deeper parts towards the surface of the defect. The extracellular matrix of the repair tissue also had more resemblance to the normal articular cartilage in this group with more intense staining compared to the control group (Figures 7(d)–7(f)).

At 24 weeks, the repair tissue in the control group had disintegrated to very large cysts containing tissue debris. The chondrocyte-like cells were only observed at the peripheral regions of the cysts and above the subchondral bone area (Figures 6(g) and 6(h)). In the PRF treated group, reparative tissue disintegration was much less than the control group although surface fissures or cracks were seen and smaller cysts had also developed but the repair tissue still consisted of chondrocyte-like cells and extracellular matrix resembling normal cartilage (Figures 7(g)–7(i)).

## 4. Discussion

The main rationale behind the use of platelet rich products in wound healing is the presence of high concentrations of different growth factors in these biological products. Mammalian wound healing consists of distinct overlapping stages of haemostasis, inflammation, proliferation, and tissue remodelling [15–17]. During the early phases of wound healing (haemostatic and inflammatory stages), platelets are attracted to the site of injury to aid in the process of haemostasis by deposition of fibrin and formation of platelet plug. Different cytokines and growth factors are also released by the platelets [17]. These bioactive products initiate and promote the process of new tissue formation [18]. Articular cartilage is avascular in nature and hence complete inflammatory response is not initiated in articular injuries. Even in full thickness injuries where inflammatory response is observed, the capacity for repair is limited leading to the formation of fibrous tissue or fibrocartilage which is structurally inferior compared to the hyaline cartilage leading to degeneration over time [19].

Growth factors are the biological products which regulate the development and homeostasis of articular cartilage throughout life [20]. Therefore the use of growth factors or products containing high concentrations of growth factors in the form of platelet rich products could be a promising form of treatment for articular cartilage regeneration. Gaissmaier et al. [8] showed that addition of 1 and 10 percent concentrations of human platelet supernatant to chondrocyte cultures led to increased cell proliferation. In a subsequent in vitro study, Akeda et al. [2] investigated the effect of platelet rich plasma on porcine chondrocyte proliferation and extracellular matrix synthesis and showed that the use of 10% PRP in culture medium led to an increase in DNA content of chondrocytes and collagen and proteoglycan synthesis. Several in vivo studies [4, 9, 13, 21–24] have also pointed out the positive effects of PRP on articular cartilage regeneration. In the in vivo studies, PRP was either used alone or in combination with other treatment methods to treat various forms of articular cartilage defects.

Choukroun's platelet rich fibrin is a second generation platelet product developed by Choukroun et al. in 2001 [25]. Autologous PRF is produced by immediate centrifugation of whole blood collected from the patient without any anticoagulants. Apart from the platelets, leukocytes are also present in this product. PRF can be used either as a clot or membrane which is produced by pressing the clot between two gauze sponges. The use of PRF has been described in oral [26], maxillofacial [27, 28], ENT (ear, nose, and throat) [29], and plastic surgery [30]. Although there are structural differences between PRF and PRP, they both contain growth factors released from the platelets.

The hypothesis of this study was that PRF could positively influence articular cartilage regeneration through the same mechanisms described for PRP. The results of macroscopic and histologic evaluations indicate that PRF use had a positive influence on cartilage repair. To the authors' knowledge, this is the first study to describe the use of Choukroun's PRF in articular cartilage repair. The only other study describing the use of Choukroun's PRF in cartilage repair is the one conducted by Kuo et al. [31]. In their study, articular cartilage defects with a diameter of 3 mm and depth of 0.5 mm were created in the weight bearing area of medial femoral condyles of right hind leg in 12 New Zealand white rabbits. The defects in 6 rabbits were filled with PRF clot mixed with the cartilage fragments obtained from the defects and ground into small 1 mm granules. The other 6 rabbits were used as control with empty defects. In all rabbits, the defects were covered by a periosteal patch obtained from the tibial shaft and sutured over the defect. The animals were euthanized three months after surgery and the results of histological grading of the repair tissue indicated significant improvement of cartilage regeneration in the PRF treated group.

In this study, full thickness articular cartilage defects were created on the femoral condyles. This specific region was chosen because of its weight bearing and due to the fact that most clinical lesions, both in humans and animals, are seen in this region. All the necessary mechanisms of cartilage repair particularly cellular migration are thought to be activated in full thickness lesions intensifying the role of growth factors in repair [23]. Focal lesions were created to limit the extent of joint damage which could negatively influence cartilage repair.

The time course for the healing of canine full thickness articular cartilage defects consists of granulation tissue formation at 1 month, cellular tissue formation with high synthetic activity and low glycosaminoglycan content at 6 weeks, metaplasia to fibrocartilage by 4 months, and imperfect hyaline cartilage formation by 6 months [32]. According to ICRS guidelines, the recommended study period for evaluation of repair tissue in animal models is between 1 to 6 months although longer periods of up to 12 months can also be used to fully evaluate the repair efficacy [33]. Different sampling times used in the present study were based on the above mentioned characteristics of canine articular cartilage healing and reflected the short (4 weeks), medium (16 weeks), and relatively long term (24 weeks) periods for the study of reparative tissue formed at the site of the defects. The results of the present study also indicated that fibrous granulation tissue had formed in the short term followed by the formation of cartilage-like repair tissue in the longer periods although the quality of repair was better in the PRF treated groups as indicated by the higher macroscopic and histological grading scores.

Although higher macroscopic and histological grading scores were observed at 24 weeks compared with the 16-week sampling time in both control and PRF treated groups but no statistically significant within-group difference was observed between the two sampling periods. The major difference between the repair tissue formed at 16 and 24 weeks was the presence of central cysts in the latter sampling time in both treatment groups. These cysts were seen in 50 and 25 percent of the control and PRF treated defects, respectively, and their presence reflected central disintegration of the repair tissue. The defects containing cysts had lower macroscopic and histological grading scores; therefore it seems that the quality of repair is inferior in the defects that contain cystic lesions at 24 weeks. The findings of Jackson et al. [34] further support our results. They created full thickness osteochondral defects of 6 mm in diameter and depth in the medial femoral condyle of adult goats and the untreated defects were allowed to heal spontaneously. Central cysts were seen inside the repair tissue at 26 and 52 weeks following the creation of defects which were attributed to gradual disintegration of the reparative tissue. In another study conducted on dogs [35], it has been shown that the histologic healing of articular cartilage consists of a proliferative phase established by 1.5 months in which a repair tissue is formed, a remodelling phase at 3 and 6 months in which articular cartilage is regenerated, and a degradation phase at 12 and 18 months in which the repair tissue and surrounding cartilage become progressively damaged. In contrast to humans, no activity restrictions are imposed on the animals and full weight bearing and mechanical loading on the defects could also be contributed to the disintegration of repair tissue. Repair tissue disintegration in PRF treated defects as evidenced by central cyst formation at 24 weeks was less than the control group which further emphasizes the positive influence of PRF on cartilage repair.

A limitation of our study was that immunohistochemical staining to specify the type of collagen produced in the repair tissue was not conducted. The other limitation was that the study period could not be extended longer, that is, up to one year to evaluate the repair tissue alterations further. Both limitations were due to financial constraints.

## 5. Conclusion

It can be concluded that the use of autologous PRF leads to macroscopic and histological improvements in articular cartilage repair and regeneration. Further studies are required to examine the effect of PRF on partial thickness and chronic articular lesions.

## Figures and Tables

**Table 1:** ICRS macroscopic evaluation of cartilage repair.

Categories	Score
Degree of defect repair	
In level with surrounding cartilage	4
75% repair of defect depth	3
50% repair of defect depth	2
25% repair of defect depth	1
0% repair of defect depth	0
Integration with border zone	
Complete integration with surrounding cartilage	4
Demarcating border <1 mm	3
3/4 of graft integrated, 1/4 with a notable border >1 mm width	2
1/2 of graft integrated with surrounding cartilage, 1/2 with a notable border >1 mm	1
From no contact to 1/4 of graft integrated with surrounding cartilage	0
Macroscopic appearance	
Intact smooth surface	4
Fibrillated surface	3
Small, scattered fissures or cracks	2
Several, small or few but large fissures	1
Total degeneration of grafted area	0
Overall repair assessment	
Grade I: normal	12
Grade II: nearly normal	11–8
Grade III: abnormal	7–4
Grade IV: severely abnormal	3–1

**Table 2:** O'Driscoll histological cartilage repair score.

Characteristics	Score
Nature of predominant tissue	
Cellular morphology	
Hyaline articular cartilage	4
Incompletely differentiated mesenchyme	2
Fibrous tissue or bone	0
Safranin-O staining of the matrix	
Normal or nearly normal	3
Moderate	2
Slight	1
None	0
Structural characteristics	
Surface regularity	
Smooth and intact	3
Superficial horizontal lamination	2
Fissures 25–100% of the thickness	1
Severe disruption including fibrillation	0
Structural integrity	
Normal	2
Slight disruption including cysts	1
Severe disintegration	0
Thickness	
100% of normal adjacent cartilage	2
50–100% of normal cartilage	1
0–50% of normal cartilage	0
Bonding to the adjacent cartilage	
Bonded at both ends of graft	2
Bonded at one end or partially at both ends	1
Not bonded	0
Freedom from cellular changes of degeneration	
Hypocellularity	
Normal cellularity	3
Slight hypocellularity	2
Moderate hypocellularity	1
Severe hypocellularity	0
Chondrocyte clustering	
No clusters	2
<25% of the cells	1
25–100% of the cells	0
Freedom from degenerative changes in adjacent cartilage	
Normal cellularity, no clusters, and normal staining	3
Normal cellularity, mild clusters, and slight staining	2
Mild or moderate hypocellularity, slight staining	1
Severe hypocellularity, poor or no staining	0
Total	**24**
